# Intake of dietary saturated fatty acids and risk of type 2 diabetes in the European Prospective Investigation into Cancer and Nutrition-Netherlands cohort: associations by types, sources of fatty acids and substitution by macronutrients

**DOI:** 10.1007/s00394-018-1630-4

**Published:** 2018-03-09

**Authors:** Shengxin Liu, Yvonne T. van der Schouw, Sabita S. Soedamah-Muthu, Annemieke M. W. Spijkerman, Ivonne Sluijs

**Affiliations:** 1 Julius Center for Health Sciences and Primary Care, University Medical Center Utrecht, Utrecht University, PO Box 85500, STR6.131, 3508 GA Utrecht, The Netherlands; 2 Division of Human Nutrition, Wageningen University and Research, Wageningen, The Netherlands; 3 Department of Medical and Clinical Psychology, Center of Research on Psychology in Somatic Disease (CORPS), Tilburg University, Tilburg, The Netherlands; 4 Centre for Nutrition, Prevention and Health Services, National Institute for Public Health and the Environment (RIVM), Bilthoven, The Netherlands

**Keywords:** Saturated fatty acids, Type 2 diabetes, Nutrition, Epidemiology, Cohort study

## Abstract

**Purpose:**

The association between dietary saturated fatty acids (SFA) intake and type 2 diabetes (T2D) remains unclear. This study aimed at investigating the association between SFA intake and T2D risk based on (1) individual SFA (differing in carbon chain length), (2) food sources of SFA and (3) the substituting macronutrients.

**Methods:**

37,421 participants from the European Prospective Investigation into Cancer and Nutrition-Netherlands (EPIC-NL) cohort were included in this study. Baseline dietary intake was assessed by a validated food frequency questionnaire. T2D risks were estimated by Cox regression models adjusted for non-dietary and dietary covariates.

**Results:**

893 incident T2D cases were documented during 10.1-year follow-up. We observed no association between total SFA and T2D risk. Marginally inverse associations were found for lauric acid (HR per 1 SD of energy%, 95% CI 0.92, 0.85–0.99), myristic acid (0.89, 0.79–0.99), margaric acid (0.84, 0.73–0.97), odd-chain SFA (pentadecylic plus margaric acids; 0.88, 0.79–0.99), and cheese derived SFA (0.90, 0.83–0.98). Soft and liquid fats derived SFA was found related to higher T2D risk (1.08, 1.01–1.17). When substituting SFA by proteins, carbohydrates and polyunsaturated fatty acids, significantly higher risks of T2D were observed (HRs per 1 energy% ranging from 1.05 to 1.15).

**Conclusion:**

In this Dutch population, total SFA does not relate to T2D risk. Rather, the association may depend on the types and food sources of SFA. Cheese-derived SFA and individual SFA that are commonly found in cheese, were significantly related to lower T2D risks. We cannot exclude the higher T2D risks found for soft and liquid fats derived SFA and for substituting SFA with other macronutrients are influenced by residual confounding by *trans* fatty acids or limited intake variation in polyunsaturated fatty acids and vegetable protein.

**Electronic supplementary material:**

The online version of this article (10.1007/s00394-018-1630-4) contains supplementary material, which is available to authorized users.

## Background

Dietary guidelines recommend that intake of total saturated fatty acids (SFA) should not exceed 10% of energy per day [1, 2]. Dietary SFA were considered to be related with increased risk of type 2 diabetes (T2D) due to previous evidence from prospective studies that found SFA associated with impaired insulin sensitivity or glucose tolerance [3–5]. However, a recent meta-analysis, which included eight prospective studies, found no association between SFA and incident T2D, with a relative risk (RR) of 0.95 (95% CI 0.88, 1.03) between extreme intake levels [6]. Evidence is accumulating that the association between SFA and T2D might depend on carbon chain length, food sources and/or the substituting macronutrients, which have not been thoroughly studied yet [7, 8].

First, individual SFA that differ in carbon chain length may have different associations with T2D risk. So far, only three studies have addressed the associations between intakes of individual SFA through self-reported dietary measurements and T2D [7, 9, 10]. These studies investigated a selection of individual SFA, and showed dissimilarities in the associations of individual SFA with T2D risk. Except for palmitic acid, the selection of SFA differed between the studies, which hampers the comparison between the studies.

Second, the association between SFA and T2D risk can be modulated by its food sources. As far as we know, no study has investigated SFA derived from different food sources in relation to T2D risk. However, a recent cross-sectional study found that SFA from meat was positively associated with more insulin resistance and less insulin secretion, whereas SFA from dairy and plant sources were beneficially related to these markers [11]. In addition, the main food sources of SFA, meat and dairy products were found to be differently associated with T2D risk. A systematic review and meta-analysis of 17 cohort studies found that meat intake was related to an increased risk of T2D [12], whereas a recent meta-analysis of 22 cohort studies found dairy products intake to be inversely related to T2D risk [13]. These findings stress the importance of clarifying the food source of SFA with regard to T2D risk.

Third, the association may also depend on macronutrients that substitute SFA in the diet. The Nurses’ Health Study and Iowa’s Women Study found that replacing energy from SFA with polyunsaturated fatty acids (PUFA) was related to lower T2D risk [14, 15]. However, no other prospective study has looked into the association between substituting SFA with macronutrients other than PUFA, and incident T2D.

The aim of this study was to investigate the associations between intake of SFA and risk of incident T2D in a Dutch population based on (1) individual SFA (differing in carbon chain length), (2) the food sources of SFA and (3) the macronutrients that substitute SFA.

## Methods

### Study population

The European Prospective Investigation into Cancer and Nutrition-Netherlands (EPIC-NL) cohort consists of the MORGEN (Monitoring Project on Risk Factors for Chronic Diseases) and the Prospect cohorts. The design and rationale of EPIC-NL were described in detail elsewhere [16]. Briefly, both cohorts were set up between 1993 and 1997 and baseline measurements were performed in 40,011 participants. The MORGEN cohort consisted of 22,654 men and women aged 20 to 59 years from three Dutch towns (Amsterdam, Doetinchem and Maastricht) as a random sample of the general population. The Prospect cohort included 17,357 women aged from 49 to 70 years, who participated in the Dutch breast cancer-screening program. Both studies complied with the Declaration of Helsinki. All participants signed informed consent before inclusion. The MORGEN was approved by the medical ethics committee of the Netherlands Organization for Applied Scientific Research (TNO), and the Prospect was approved by the institutional review board of the University Medical Centre Utrecht.

For this study, participants were excluded when one of the following criteria was fulfilled: participants who withheld permission for linkage with disease registries (*n* = 1046); prevalent diabetes cases at baseline (*n* = 679); participants with missing information of dietary intake (*n* = 180) and other co-variables, such as education information and physical activity level, (*n* = 339); participants with implausible energy intake based on the ratio of reported energy intake to estimated basal metabolic rate, i.e., the top or bottom 0.5% of the ratio (*n* = 346); leaving a total of 37,421 men and women for analyses.

### Measurement of food intake, saturated fatty acids and other nutrients

Food intake was assessed by a self-administered Food Frequency Questionnaire (FFQ) on the usual consumption frequency of 79 main food categories during the year preceding enrolment. The FFQ was described in detail elsewhere [17]. The FFQ was validated against 12 24-h recalls between both genders before the start of the study [18]. Spearman’s correlation coefficients for intake of total SFA and individual SFA ranged from 0.53 to 0.71 in men and from 0.30 to 0.66 in women [19].

The intakes for all micronutrients and macronutrients except individual SFA were calculated based on the Dutch food composition table 1996 [20]. The intake of individual SFA was calculated from the Dutch food composition table 1998 (digital update). Using the analogy of a previous research [19], seven independent predefined food groups were identified which together contribute > 82% of the mean total SFA intake in the study population: butter, cheese, milk and milk products, meat, cakes, snacks and fats. The group of fats was separated into two subgroups based on their SFA content: hard and solid fats (including margarines and fats in wrappers and solid frying fats, all of which contained ≥ 20 g SFA/100 g of the product) and soft and liquid fats (including soft margarines, vegetable oils, liquid fats, and frying oils, all of which contained < 20 g SFA/100 g of the product). The remaining food groups were aggregated and labelled as the others.

Intake of total SFA was calculated as the sum of SFA derived from the predefined food groups mentioned above. Considering relative low intakes of single individual SFA, two additional groups were made to add efficiency of the study: short- and medium-chain SFA (sum of butyric, caproic, caprylic and capric acids) and odd-chain SFA (pentadecylic plus margaric acids).

Energy reporting status of participants was estimated by comparison between self-reported energy intake and BMR (Basal metabolic rate, which was calculated using the Schofield equation [21]). According to the Goldberg cut-offs [22], participants with an energy intake vs. BMR of < 1.14 were defined as energy under-reporters, whereas those with an energy intake vs. BMR of > 2.40 were classified as energy over-reporters. Both under-reporters and over-reporters were defined as mis-reporters, while remaining participants were defined as normal energy-reporters.

Alcohol consumption was categorized as follows: 0, 0.1–6.0, 6.1–12.0, 12.1–24.0, and > 24.0 g/day for women and 0, 0.1–6.0, 6.1–12.0, 12.1–24.0, 24.1–60.0, and > 60.0 g/day for men. Glycaemic index (GI) of foods, a measure of the extent to which foods raise the blood glucose level, was obtained from international table complied by Foster-Powell et al. [23]. To adjust for total energy intake, intake of total SFA, individual SFA, SFA from specific food groups, or other macronutrient intake was calculated and expressed as nutrient density, and micronutrients were adjusted through use of the residual method [24].

### Ascertainment and verification of diabetes

Three sources of ascertaining incidence T2D were included in this study: self-report, hospital discharge diagnoses and urinary glucose strip tests (only among the Prospect participants, 1.6% of total potential diabetes cases). Verification of potential diabetes cases detected by any of these methods was carried out with information from participants’ general practitioner or pharmacist. Verification information was available for 89% of the potential diabetes cases, and 72% of these cases were verified as T2D and subsequently used for the analysis. The process of ascertainment and verification of incident and prevalent diabetes cases is described in detail elsewhere [25].

### Other measurements

At baseline, information on demographic characteristics, lifestyle characteristics, the presence of chronic diseases, and risk factors of chronic diseases was obtained with general questionnaires. Smoking status was categorized as current, past or never smoked. Family history of diabetes was defined as at least one parent with diabetes. Education was classified as low, middle and high education. Physical activity was measured with a validated questionnaire, from which the Cambridge Physical Activity Index was calculated and categorized as inactive, moderately inactive, moderately active and active [26].

A baseline physical examination was performed according to standard operating procedures. Body weight, height, waist circumference and blood pressure were measured. BMI was calculated as weight divided by height squared (kg/m^2^). Systolic and diastolic blood pressures were obtained as the mean of two measurements in the supine position on the left arm using a random zero sphygmomanometer (MORGEN), or on the right arm using a BOSO Oscillomat (Bosch & Son, Jungingen, Germany) (Prospect). Hypertension was defined when one or more of the following criteria were fulfilled: diastolic blood pressure > 90 mmHg, systolic blood pressure > 140 mmHg, self-reported presence of hypertension or use of hypertensive medication. Hyperlipidaemia was defined through self-reported diagnosis and/or use of medication.

### Data analysis

Baseline characteristics of the study population were calculated across quartiles of total SFA intake in percentage of energy and presented as means (standard deviation) and median (interquartile range) for continuous variables, or percentage for categorical variables. Correlations between dietary intakes of total SFA, individual SFA and SFA from different food sources, as well as correlations between intakes of SFA from different food sources, total fatty acids from that food source and the food source itself, were calculated using Pearson correlations coefficients.

Person-years were calculated as the time between the date of the study entry and the dates of diabetes diagnosis, death, loss to follow-up or end of follow-up (1 January 2006), whichever came first. The endpoint of analysis was incident T2D.

Cox proportional hazard regression models were used to calculate HRs with 95% CIs for the association between SFA intake and risk of T2D. Total SFA intake was evaluated per 1% of energy and entered as a continuous variable into Cox regression models. In addition to a crude model which was adjusted for total energy intake (Model 1), four models were constructed to adjust for potential confounding. Model 2 was adjusted for sex (men or women) and age at baseline (continuous). Model 3 was additionally adjusted for lifestyle and demographic factors: alcohol consumption level (six categories), smoking (three categories), physical activity (four categories), and education level (three categories). Model 4 was additionally adjusted for dietary factors (continuous): energy-adjusted intake of *trans* fatty acids, protein (both animal- and vegetable-), dietary cholesterol, vitamin E and fibre. Model 5 was additionally adjusted for BMI (continuous) and waist circumference (continuous). Similar models were used for intake of SFA in grams per day. Intakes of individual SFA or SFA from specific food sources were separately evaluated per 1 SD of intake and entered into Cox models as continuous variables. We used the same adjustment models as described above, except that we additionally adjusted for the sum of all other consumed SFA.

Cox models were converted into substitution models to estimate the risk of T2D when energy intake from total SFA was substituted with an equal amount of energy from each of the other macronutrients (PUFA, monounsaturated fatty acids (MUFA), *trans* fatty acids, total carbohydrates, animal protein, and vegetable protein) [27]. We choose to model the substitutions per 1 en% as this best matched with the level and variation of intake of the macronutrients. Total energy intake from all macronutrients except energy from alcohol consumption was also included. By excluding SFA intake from the models, the HR for each macronutrient can be interpreted as the difference in T2D risk for each additional intake of 1% of energy from that particular macronutrient at the expense of 1% of energy from SFA. Additionally, the analysis for substitution with total carbohydrates was stratified for tertiles of the GI distribution, to differentiate in carbohydrate quality. In this way, the substitution of SFA with carbohydrates in GI tertiles 1, 2, and 3 represented the substitution of SFA with carbohydrates in participants with a low-, medium-, and high-GI diet, respectively.

The proportional hazards assumption was tested by calculating Schoenfeld residuals and visual inspection of log–log plots, no deviation was detected. Interactions of total SFA intake (en%) with BMI categories (< 25.0, 25.0–29.9 and ≥ 30.0 kg/m^2^), family history of T2D (without family history and with family history), energy reporting status (mis-reporters and normal energy-reporters) and sex (men and women) were estimated using a likelihood ratio test and by including continuous interaction terms in the multivariable adjusted model (model 5). The analysis was repeated in the same population after excluding the first 2 years of follow-up to minimize the possibility of reverse causation. The analysis was also performed in the population after excluding prevalent cases of cancer or cardiovascular disease or hypertension or hyperlipidaemia and participants who had a stroke or myocardial infarction, due to their possible dietary changes caused by the illness. Furthermore, in additional analyses, we estimated the associations between non-SFA fatty acids derived from different food sources (per 1 SD of intake) and T2D risk, to see whether we can actually tease SFA apart from other fatty acids derived from the same food source in relation with T2D.

All statistical analyses were executed in SAS 9.4 (SAS Institute), and P-values < 0.05 (2-sided) was considered as statistically significant.

## Results

### Baseline characteristics

In this study population (mean age 49 years, 74.4% women), participants with a relatively high SFA intake were on average older women with a lower education level and were more likely to be a smoker and less devoted to physical activity. They also tended to have a higher BMI and waist circumference, a higher blood pressure, but were less likely to have hyperlipidaemia (Table 1). Furthermore, participants with high SFA intake reported higher intakes of MUFA, *trans* fatty acids, animal protein, cholesterol, vitamin E, vitamin D and calcium, and lower intakes of vegetable protein, carbohydrates, fibre and alcohol. Dietary GI and intake of PUFA were similar across SFA intake levels.

**Table 1:** Baseline characteristics across quartiles of saturated fatty acid intake (en%) in 37,421 participants from the EPIC-NL cohort

Median (intake range), en%	Q1: 10.9(2.1–12.3)	Q2: 13.2(12.3–13.9)	Q3:14.7(13.9–15.6)	Q4:17.3(15.6–27.1)
Subject, *n*	9355	9355	9356	9355
Incident T2D cases, *n*	198	218	223	254
Age, year	48 ± 13	48 ± 12	49 ± 12	52 ± 11
Male, %	28.6	29.3	24.8	19.7
High educational level^a^, %	24.3	22.5	19.9	16.1
High physical activity^b^, %	42.4	43.3	41.9	39.6
Current smoker, %	29.2	29.4	30.1	33.5
High alcohol consumer^c^, %	23.6	15.9	11.5	8.4
Family history of T2D, %	17.0	17.1	18.7	19.3
Mis-reporters of energy intake^d^, %	34.4	24.9	21.6	21.0
BMI, kg/m^2^	25.3 ± 3.8	25.5 ± 3.8	25.7 ± 4.0	26.0 ± 4.2
Waist circumference, cm	84.3 ± 11.1	84.9 ± 11.3	85.2 ± 11.3	85.9 ± 11.8
Hypertension^e^, %	37.4	35.3	36.0	38.5
Hyperlipidaemia, %	12.3	8.3	6.8	5.5
Energy intake, kcal	1923.3 ± 593.5	2075.2 ± 607.9	2099.9 ± 597.7	2112.5 ± 611.8
Saturated fatty acid intake, g/day	23.4 ± 8.0	30.4 ± 9.0	34.4 ± 9.9	40.6 ± 12.3
Saturated fatty acid intake, en%	10.9 ± 1.2	13.2 ± 0.5	14.7 ± 0.5	17.3 ± 1.5
Lauric acid (12:0), en%	0.5 ± 0.2	0.6 ± 0.2	0.7 ± 0.2	0.8 ± 0.3
Myristic acid (14:0), en%	1.1 ± 0.3	1.4 ± 0.3	1.6 ± 0.3	2.0 ± 0.4
Pentadecylic acid (15:0), en%	0.2 ± 0.0	0.2 ± 0.1	0.2 ± 0.1	0.3 ± 0.1
Palmitic acid (16:0), en%	5.8 ± 0.8	6.7 ± 0.6	7.4 ± 0.6	8.5 ± 0.9
Margaric acid (17:0), en%	0.1 ± 0.0	0.2 ± 0.0	0.2 ± 0.0	0.2 ± 0.0
Stearic acid (18:0), en%	2.8 ± 0.5	3.3 ± 0.4	3.7 ± 0.4	4.2 ± 0.5
Sum of butyric (4:0) through capric (10:0) acid, en%	0.5 ± 0.2	0.6 ± 0.2	0.7 ± 0.2	0.9 ± 0.3
Sum of pentadecylic (15:0) and margaric (17:0) acid, en%	0.3 ± 0.1	0.4 ± 0.1	0.4 ± 0.1	0.5 ± 0.1
SFA from food source, g/day				
Butter	0.8(0.4–1.5)	1.3(0.7–2.2)	1.6(0.9–3.0)	2.5(1.3–6.4)
Cheese	3.1(1.5–4.8)	4.2(2.6–6.7)	5.4(3.3–7.9)	6.9(3.9–10.5)
Milk and milk products	3.4(1.9–5.3)	4.6(2.8–6.7)	5.1(3.2–7.5)	5.6(3.4–8.3)
Meat	3.8(2.1–5.8)	5.0(3.1–7.2)	5.7(3.5–7.8)	6.1(3.8–8.7)
Cakes and cookies	1.1(0.5–2.1)	1.6(0.8–2.8)	1.9(1.0–3.2)	2.0(1.0–3.3)
Snacks	0.5(0.2–1.2)	0.7(0.3–1.4)	0.7(0.3–1.4)	0.6(0.2–1.1)
Hard, solid fat	1.0(0.3–2.1)	1.8(0.8–3.3)	2.5(1.1–4.5)	3.7(1.7–6.5)
Soft, liquid fat	1.8(1.0–2.9)	2.0(1.2–3.2)	1.9(1.1–3.1)	1.5(0.7–2.6)
Others	4.8(3.3–6.8)	5.3(3.8–7.7)	5.4(3.8–7.7)	5.0(3.5–7.3)
PUFA, en%	6.3 ± 1.8	6.5 ± 1.7	6.5 ± 1.6	6.3 ± 1.6
MUFA, en%	8.2 ± 1.8	9.3 ± 1.6	10.0 ± 1.6	11.0 ± 1.8
*trans* Fat, en%	1.1 ± 0.4	1.3 ± 0.4	1.5 ± 0.4	1.7 ± 0.5
Animal protein, en%	9.2 ± 2.8	9.7 ± 2.5	10.0 ± 2.3	10.6 ± 2.4
Vegetable protein, en%	6.0 ± 1.2	5.7 ± 0.9	5.5 ± 0.9	5.1 ± 0.8
Carbohydrates, en%	49.2 ± 6.8	46.4 ± 5.3	44.4 ± 4.9	41.1 ± 5.0
Fibre, g/day	24.8 ± 5.6	23.7 ± 4.6	23.0 ± 4.3	21.8 ± 4.2
Cholesterol, g/day	12.4 ± 3.5	12.4 ± 3.2	12.3 ± 3.1	11.8 ± 3.1
Vitamin E, mg/day	185.2 ± 54.8	208.8 ± 51.8	224.1 ± 51.6	250.6 ± 57.6
Vitamin D, mg/day	2.4 ± 0.9	2.7 ± 0.8	3.0 ± 0.8	3.2 ± 0.9
Calcium, mg/day	1004.3 ± 370.7	1040.2 ± 341.2	1078.0 ± 336.5	1139.1 ± 352.8

*en%* percentage of energy, *EPIC-NL* European Prospective Investigation into Cancer and Nutrition-Netherlands, *Q* quartiles

^a^Low: primary education up to completing intermediate vocational education; middle: up to higher secondary education; high: those with higher vocational education and university

^b^Categorized according to Cambridge Physical Activity Index (CPAI)

^c^High: women who consume > 24 g/day and men who consume > 60 g/day

^d^Mis-reporters of energy intake: subjects with an energy intake vs. BMR < 1.14 or > 2.40

^e^Defined by a physician-diagnosed self-report, measured hypertension (> 140 systolic or > 90 diastolic) or use of hypertensive medication

The mean ± SD baseline intake of total SFA in the study population was 14.0 ± 2.6 percentage of energy. More than 95% of the population surpassed the upper intake limit of 10% of energy per day recommended by the Health Council of the Netherlands. The long-chain SFA palmitic acid (51.1%) and stearic acid (25.2%) represented most of the SFA intake (Supplemental Fig. 1). Cheese (17.0%), meat (16.9%), milk and milk products (15.8%) were the main food sources of SFA (Supplemental Fig. 2). High Pearson’s correlations were observed between palmitic and stearic acids (*r* = 0.92), myristic and pentadecylic acids (*r* = 0.92), myristic and margaric acids (*r* = 0.85), pentadecylic and margaric acids (*r* = 0.88) (Supplemental Table 1). SFA from specific food source was highly correlated with total fatty acids from that food source, as well as the intake of the food source itself (Supplemental Tables 2 and 3).

### Total SFA intake and T2D risk

During a median follow-up of 10.1 years, 893 incident T2D cases were documented. After multivariable adjustment for lifestyle and demographic factors, dietary factors, BMI and waist circumference (model 5), no significant association between intake of energy from total SFA with risk of T2D was observed (SFA_total_ HR _per 1% of energy_: 0.97; 95% CI 0.94, 1.00). This result was similar when intake of SFA was modelled in grams per day (SFA_total_ HR _per 1 g/d_: 1.00; 95% CI 0.98, 1.01) (Table 2).

**Table 2:** Multivariable HRs with 95% CIs for the association between the intake of total SFA and incidence of type 2 diabetes in 37,421 participants from the EPIC-NL cohort

	Median intake	Model 1^c^	Model 2^d^	Model 3^e^	Model 4^f^	Model 5^g^
Total SFA per 1 en%^a^	14 en%/day	1.05 (1.03, 1.08)	1.02 (1.00, 1.05)	1.00 (0.97, 1.03)	0.96 (0.93, 0.99)	0.97 (0.94, 1.00)
Total SFA per g^b^	32 g/day	1.03 (1.02, 1.04)	1.02 (1.00, 1.03)	1.00 (0.99, 1.02)	0.99 (0.98, 1.01)	1.00 (0.98, 1.01)

Obtained from Cox proportional hazards regression models. EPIC-NL, European Prospective Investigation into Cancer and Nutrition-Netherlands

^a^en%, percentage of energy. Total SFA intake was evaluated as en% and entered as a continuous variable

^b^Total SFA intake was evaluated as g/day and entered as a continuous variable

^c^Crude model; adjustment for total energy intake

^d^Adjustments for sex, age

^e^Additional adjustments for education level, energy-adjusted alcohol consumption level, smoking status, physical activity level

^f^Additional adjustments for energy-adjusted intake of animal protein, vegetable protein, *trans* fatty acids, vitamin E, dietary fibre and cholesterol

^g^Additional adjustments for BMI and waist circumference

### Intake of individual SFA and risk of T2D

Table 3 shows the HRs for the association between individual SFA differing in carbon chain length and risk of T2D. After multivariable adjustment (model 5), significantly lower T2D risks were observed for each additional SD of intake of energy from lauric acid (HR_per SD increase_: 0.92; 95% CI 0.85, 0.99), myristic acid (HR_per SD increase_: 0.89; 95% CI 0.79, 0.99), margaric acid (HR_per SD increase_: 0.84; 95% CI 0.73, 0.97) and odd-chain SFA (pentadecylic plus margaric acids; HR_per SD increase_: 0.88; 95% CI 0.79, 0.99). No significant associations were observed for intakes of short- and medium-chain SFA (butyric through capric), palmitic, pentadecylic and stearic acids.

**Table 3:** Multivariable HRs with 95% CIs for the associations between the intakes of individual SFA (en %) and incidence of type 2 diabetes in 37,421 participants from the EPIC-NL cohort

	Median intake (en%/day)	SD^a^	Model 1^b^	Model 2^c^	Model 3^d^	Model 4^e^	Model 5^f^
Lauric acid (12:0)	0.63	0.24	1.03 (0.97, 1.10)	0.88 (0.82, 0.94)	0.89 (0.83, 0.95)	0.89 (0.83, 0.97)	0.92 (0.85, 0.99)
Myristic acid (14:0)	1.51	0.45	1.04 (0.97, 1.10)	0.89 (0.83, 0.96)	0.91 (0.85, 0.97)	0.78 (0.70, 0.87)	0.89 (0.79, 0.99)
Pentadecylic (15:0)	0.21	0.08	1.01 (0.94, 1.08)	0.89 (0.82, 0.95)	0.92 (0.86, 0.99)	0.79 (0.71, 0.88)	0.90 (0.80, 1.00)
Palmitic acid (16:0)	7.06	1.22	1.08 (1.02, 1.16)	1.05 (0.99, 1.12)	1.05 (0.98, 1.11)	0.95 (0.84, 1.08)	0.90 (0.80, 1.03)
Margaric acid (17:0)	0.16	0.04	1.09 (1.02, 1.17)	1.00 (0.93, 1.07)	1.02 (0.95, 1.09)	0.76 (0.66, 0.87)	0.84 (0.73, 0.97)
Stearic acid (18:0)	3.47	0.68	1.09 (1.02, 1.16)	1.06 (0.99, 1.13)	1.04 (0.98, 1.11)	1.01 (0.90, 1.12)	0.92 (0.83, 1.03)
Sum of butyric (4:0) to capric (10:0) acids	0.67	0.28	1.00 (0.94, 1.07)	0.87 (0.81, 0.94)	0.91 (0.85, 0.98)	0.86 (0.79, 0.94)	0.93 (0.85, 1.01)
Sum of pentadecylic (15:0) and margaric(17:0) acids	0.37	0.11	1.04 (0.97, 1.10)	0.93 (0.87, 0.99)	0.96 (0.89, 1.02)	0.78 (0.69, 0.87)	0.88 (0.79, 0.99)

Obtained from Cox proportional hazards regression models. EPIC-NL, European Prospective Investigation into Cancer and Nutrition-Netherlands

^a^HRs are expressed per SD en%

^b^Crude model; adjusted for total energy intake

^c^Adjusted for sex, age and sum of other SFA

^d^Additional adjustments for education level, energy-adjusted alcohol consumption level, smoking status, physical activity level

^e^Additional adjustments for energy-adjusted intake of animal protein, vegetable protein, *trans* fatty acids, vitamin E, dietary fibre and cholesterol

^f^Additional adjustments for BMI and waist circumference

### Intake of SFA from different food sources and risk of T2D

A significant inverse association was observed between intake of cheese derived SFA and risk of T2D (HR_per SD increase_: 0.90; 95%I 0.83, 0.98) after multivariable adjustment (model 5), but not for milk and milk products derived SFA (HR_per SD increase_: 1.01; 95% CI 0.93,1.10). Higher intake of soft and liquid fats derived SFA was associated with a higher risk of T2D (HR_per SD increase_: 1.08; 95%CI 1.01, 1.17). No significant associations were observed for intake of SFA from other food sources (Table 4).

**Table 4:** Multivariable HRs with 95% CIs for the associations between intake of SFA from different food sources (en %) and incidence of type 2 diabetes in 37,421 participants from the EPIC-NL cohort

SFA from:	Median intake en%/day	SD^a^	Model 1^b^	Model 2^c^	Model 3^d^	Model 4^e^	Model 5^f^
Butter	0.67	1.44	0.96 (0.89, 1.03)	0.88 (0.82, 0.95)	0.90 (0.84, 0.97)	0.93 (0.86, 1.01)	0.93 (0.86, 1.01)
Cheese	2.33	1.98	0.93 (0.87, 1.00)	0.89 (0.83, 0.95)	0.94 (0.88, 1.01)	0.86 (0.79, 0.93)	0.90 (0.83, 0.98)
Milk and milk products	2.30	1.47	1.10 (1.03, 1.17)	1.02 (0.95, 1.09)	0.99 (0.92, 1.05)	0.96 (0.89, 1.04)	1.01 (0.93, 1.10)
Meat	2.53	1.47	1.22 (1.15, 1.29)	1.23 (1.17, 1.31)	1.20 (1.13, 1.28)	1.10 (1.02, 1.19)	1.03 (0.95, 1.11)
Cakes and cookies	0.81	0.84	1.02 (0.95, 1.08)	0.88 (0.82, 0.95)	0.89 (0.83, 0.96)	0.94 (0.87, 1.01)	0.97 (0.90, 1.05)
Snacks	0.30	0.40	0.71 (0.65, 0.77)	1.00 (0.92, 1.09)	1.01 (0.93, 1.10)	0.96 (0.87, 1.06)	0.90 (0.81, 1.00)
Hard, solid fats	1.02	1.27	1.13 (1.07, 1.20)	1.05 (0.99, 1.12)	0.98 (0.92, 1.05)	0.96 (0.89, 1.04)	0.98 (0.91, 1.06)
Soft, liquid fats	0.59	0.51	1.16 (1.09, 1.23)	1.11 (1.04, 1.18)	1.12 (1.06, 1.19)	1.08 (0.99, 1.16)	1.08 (1.01, 1.17)
Others	2.57	1.08	0.79 (0.73, 0.85)	0.98 (0.90, 1.05)	1.00 (0.92, 1.08)	0.95 (0.86, 1.06)	0.96 (0.87, 1.06)

Obtained from Cox proportional hazards regression models. EPIC-NL, European Prospective Investigation into Cancer and Nutrition-Netherlands

^a^HRs are expressed per SD en%

^b^Crude model; adjusted for total energy intake

^c^Adjustment for sex, age and sum of other SFA

^d^Additional adjustments for education level, energy-adjusted alcohol consumption level, smoking status, physical activity level

^e^Additional adjustments for energy-adjusted intake of animal protein, vegetable protein, *trans* fatty acids, vitamin E, dietary fibre and cholesterol

^f^Additional adjustments for BMI and waist circumference

### Substitution of SFA by other macronutrients

Table 5 presents risks of T2D for each additional intake of 1% of energy from vegetable protein, animal protein, MUFA, PUFA or carbohydrates at the expense of equal amounts of energy from SFA. After multivariable adjustment (model 5), we found that substituting SFA with animal protein (HR _per 1% of energy_: 1.12; 95% CI 1.07, 1.17) or carbohydrates (HR _per 1% of energy_: 1.05; 95% CI 1.02, 1.08) was associated with a significantly higher risk of developing T2D. Surprisingly, we also observed an increased T2D risk when substituting SFA with vegetable protein (HR _per 1% of energy_: 1.15; 95% CI 1.03, 1.28) or PUFA (HR _per 1% of energy_: 1.15; 95% CI 1.04, 1.27). Substituting SFA with carbohydrates differing in GI values resulted in differences in T2D risk: substitution with high GI carbohydrates associated with a higher risk of T2D (HR _per 1% of energy_: 1.12; 95% CI 1.06, 1.19), whereas substitution with low- or medium GI carbohydrates did not relate to T2D risk. No significant association was observed with substitution of SFA with MUFA.

**Table 5:** Multivariable HRs with 95% CIs for the association between the consumption of 1% of energy from different macronutrients at the expense of 1% of energy from total SFA while keeping total energy intake constant and incident type 2 diabetes in 37,495 participants from the EPIC-NL cohort

	Model 1^a^	Model 2^b^	Model 3^c^	Model 4^d^	Model 5^e^
Vegetable protein	1.02 (0.94, 1.10)	1.09 (1.00, 1.18)	1.12 (1.04, 1.22)	1.20 (1.07, 1.33)	1.15 (1.03, 1.28)
Animal protein	1.20 (1.16, 1.24)	1.21 (1.17, 1.25)	1.22 (1.17, 1.28)	1.21 (1.16, 1.27)	1.12 (1.07, 1.17)
MUFA	1.03 (0.98, 1.08)	1.09 (1.03, 1.14)	1.11 (1.05, 1.18)	1.09 (1.03, 1.16)	1.04 (0.98, 1.11)
PUFA	1.19 (1.14, 1.25)	1.20 (1.15, 1.25)	1.21 (1.15, 1.27)	1.20 (1.08, 1.33)	1.15 (1.04, 1.27)
Carbohydrates	1.03 (1.01, 1.05)	1.05 (1.03, 1.07)	1.06 (1.03, 1.09)	1.07 (1.04, 1.10)	1.05 (1.02, 1.08)
Carbohydrates with a low GI^f^	1.04 (1.01, 1.07)	1.04 (1.01, 1.07)	1.04 (1.00, 1.09)	1.03 (0.98, 1.08)	1.01 (0.97, 1.06)
Carbohydrates with a medium GI^f^	1.00 (0.97, 1.04)	1.02 (0.98, 1.06)	1.01 (0.96, 1.07)	1.02 (0.97, 1.08)	1.00 (0.95, 1.06)
Carbohydrates with a high GI^f^	1.05 (1.01, 1.09)	1.11 (1.06, 1.15)	1.13 (1.08, 1.20)	1.15 (1.09, 1.22)	1.12 (1.06, 1.19)

Obtained from Cox proportional hazards regression models. EPIC-NL, European Prospective Investigation into Cancer and Nutrition-Netherlands

^a^Crude substitution model; adjustment for total energy intake

^b^Adjustment for sex and age

^c^Additional adjustments for education level, energy-adjusted alcohol consumption level, smoking status, physical activity level

^d^Additional adjustments for energy-adjusted intake of *trans* fatty acids, vitamin E, dietary fibre and cholesterol

^e^Additional adjustments for BMI and waist circumference

^f^Number of cases for low GI: 322; medium GI: 290; and high GI: 281

### Sensitivity analysis

No significant effect modification of family history (*P* values: 0.13–0.99) and energy reporting status (*P* values: 0.20–0.99) was found. Moreover, no meaningful effect modifications by baseline BMI and sex were observed for the relations of SFA and T2D (for all three approaches: individual SFA, SFA from food groups, and substitution with other macronutrients), but interactions existed between sex and substituting SFA with animal protein and PUFA (*P* values all < 0.01). Stratification for sex in substitution models indicated a significant higher risk of T2D in women when substituting SFA with animal protein or PUFA, whereas this association was not present in men (Supplemental Table 4). Our results did not materially change after excluding the first 2 years of follow-up (SFA_total_ HR _per 1% of energy_: 0.97; 95% CI 0.93, 1.00), or restricting analyses by including only baseline healthy participants (SFA_total_ HR _per 1% of energy_: 1.01; 95% CI 0.94, 1.08). Associations between non-SFA fatty acids from specific food sources and T2D risk were fairly similar to associations between SFA from that food sources and T2D risk (Supplemental Table 5).

## Discussion

In this large prospective cohort study of 37,421 participants, we found that the association between dietary SFA and risk of T2D is partly depending on the types and food sources of SFA. While no association was found for dietary total SFA, inverse associations with T2D were observed for dietary lauric acid, myristic acid, margaric acid and sum of odd-chains SFA, and cheese derived SFA. We also found a modestly higher T2D risk for soft and liquid fats derived SFA, and for substituting total SFA with proteins, carbohydrates or PUFA.

Strengths of this study include the prospective design which minimized possible recall bias, and a high follow-up retention rate of 97.6% which reduced bias caused by loss to follow-up. In addition, verification of ascertained cases of T2D diminished the presence of false-positive cases and increased validity of outcome measurement, although misclassification could still remain due to no verification of self-reported non-diabetic participants [28]. Several limitations need to be addressed. First of all, the information of dietary intake, including SFA intake, was measured with one FFQ at baseline and without repeated assessments. The FFQ relies on self-report and is prone to imprecision and reporting bias. Additionally, we were not able to take into account changes in dietary intake during follow-up, which might have diluted the observed associations. Moreover, the intake of SFA was on average high, with little variation between individuals. This may have limited us in detecting associations of SFA with T2D. Also, the high correlations of intake of SFA from specific food sources with the total fatty acids from that food sources and the food source itself suggest that we were unable to distinguishing food-derived SFAs from its food source in relation to T2D risk. This was also confirmed in the additional analyses, where we modelled non-SFA fatty acids from different food source and risk of T2D. Associations with T2D risk for non-SFA fatty acids from specific food sources were all similar to those for SFAs from that food sources. Furthermore, although all possible available confounders were corrected for, we cannot exclude the possibility of influences from residual confounding, such as cholesterol-lowering medication during follow-up. It could be hypothesized that participants with higher intake of SFA will have a higher cholesterol levels and are more eligible to take cholesterol-lowering medication [29]. In the Doetinchem cohort, a sub-cohort of EPIC-NL which was examined every 5 years, a noticeable increase of cholesterol-lowering medication usage was observed over time [30]. Evidence from a collaborative meta-analysis of clinical trials showed that cholesterol-lowering medication is associated with higher risk of T2D [31]. This may have partly affected our findings towards neutral. Finally, due to the many analyses that were performed in this study, we cannot exclude chance findings.

In line with a recent meta-analysis of eight prospective studies [6], we observed no association between dietary intake of total SFA and T2D risk. This lack of association no longer held when the carbon chain length of individual SFA was taken into account. Although three previous studies have examined the association of dietary individual SFA with T2D risk, only a selection of individual SFA was included [7, 9, 10]. We evaluated a more complete profile of individual SFA. We observed that dietary lauric and myristic acids were inversely associated with T2D risk, whereas dietary palmitic and stearic acids were not related to T2D risk. These findings are in line with the Malmö Diet and Cancer (MDC) cohort study [7]. In the MDC cohort study, dietary intake of SFA with 4–10 carbons was inversely associated with T2D risk, whereas the present study observed no association for these SFA with T2D after adjustment for BMI and waist circumference. In the MDC cohort study, the association after adjustment for BMI was also attenuated, but remained statistically significant. Differences in findings may be due to a smaller variation of intake of SFA with 4–10 carbons in our study population (IQR 0.5–0.8 energy percentage) compared to the MDC study (IQR 0.7–2.6 energy percentage). Additionally, whether overweight is a confounder or mediator in the association between SFA and T2D still remains unclear, and we cannot exclude possible over-adjustment by overweight in our study. This also applies to the association of pentadecylic acid with T2D which attenuated and became non-significant after adjustment for BMI and waist circumference. A case-cohort study of 3737 men and women also observed no association for dietary palmitic, stearic, and pentadecylic acids, which further supports our findings [10]. Any comparison between our study and the third study, a case-cohort study among participants of the EPIC-Norfolk study, is limited due to differences in adjustments for dietary factors such as *trans* fatty acids and cholesterol [9]. None of these three previous studies have investigated dietary intake of margaric acid and odd-chain SFA (pentadecylic plus margaric acids), which we found were inversely associated with T2D risk. These findings are consistent with the existing evidence from the EPIC-InterAct study, where plasma phospholipid odd-chain SFA, which are reasonable biomarkers for dietary intake of odd-chain SFAs, were inversely related to T2D risk in the InterAct study that included participants from our cohort [8].

The associations we observed for individual SFA correspond to our findings for SFA derived from different food sources. To our knowledge, this is the first observational study that investigated SFA from different food sources in relation to incident T2D. We observed that dietary SFA derived from cheese was inversely related to T2D risk. This finding is supported by previous studies that found intake of cheese to be associated with lower T2D risk [7, 32], although not consistently [13]. Given the high correlations of cheese and cheese-derived SFA (*r* = 0.98, Supplemental Table 3), it is difficult to conclude whether the observed inverse association of cheese derived SFA is due to (particular) SFA, cheese as a whole product, or other constituents present in cheese such as calcium [33]. Moreover, we did not observe any association with SFA derived from milk and milk products, despite that SFA types present in milk are similar to cheese. This could be due to the lower SFA content in milk and milk products (and lower variation in intake compared to cheese SFA), or due to differences in food matrices and fermentation of cheese and milk (products) [34]. Additional adjustments for two cheese-constituents, calcium and vitamin D, did not alter the associations of SFA from cheese with T2D, but this does not fully exclude the possibility that we are looking at more than SFA in cheese. Also, we did not observe any association with SFA derived from butter. These findings are consistent with the Netherlands Epidemiology of Obesity (NEO) study, which suggests that SFA from butter or milk is not associated with glucose–insulin metabolism [11]. Additionally, this is in agreement with recent meta-analyses that reported no associations between butter, milk and milk products and T2D [13, 35]. Our observed harmful association for meat derived SFA (HR_per SD increase_: 1.10; 95% CI 1.02, 1.19) is also in line with existing evidence [11, 36]. When we additionally adjusted for BMI and waist circumference, however, the observed association attenuated and became non-significant (HR_per SD increase_: 1.03; 95% CI 0.95, 1.11).

As far as we know, no previous studies have investigated substitutions of SFA with protein, carbohydrate nor MUFA in relation to T2D risk. We found substituting SFA with carbohydrate, animal protein, vegetable protein or PUFA was associated with higher T2D risk, but not with MUFA. Previous studies suggest that animal protein is related to a higher T2D risk [37–39], which supported our observations. Several cohort studies linked total carbohydrates to a higher risk of T2D [40–42], while others suggested a non-significant inverse association [43–45]. Nevertheless, our analysis suggests that either animal protein or total carbohydrates is worse than SFA with respect to T2D risk. Perhaps, this is because a large part of SFA consumed by our study population was derived from dairy products, and those seem to be beneficial with respect to T2D risk [13]. This might also have contributed to the finding that substituting vegetable protein for SFA related to higher T2D risk. Moreover, the small range of intake in vegetable protein may have contributed to this finding. Yet, it remains unclear why substituting SFA with PUFA was related to a higher T2D risk in our study. As suggested by previous studies [14, 15], such substitution was expected to be inversely associated with T2D risk. A possible explanation for this unexpected finding is the food sources of SFA and PUFA in this study population. Margarines, as a food source of PUFA, also contained *trans* fatty acids at the beginning of the study. During the mid-1990s, there was a notable reduction of *trans* fatty acids content in margarines. This may have led to residual confounding by underestimated *trans* fatty acids intake, consequently affecting the observed association of substituting SFA with PUFA. This may also explain why we found higher T2D risk with SFA from soft and liquid fats. Furthermore, compared to the Nurses’ Health Study (80% central range 2.9–6.2 energy percentage), the intake range of PUFA is relatively small in our study (80% central range 4.9–7.6 energy percentage). As we only found adverse association for substituting SFA with animal protein or PUFA in women in the gender-stratified analysis, the result could be largely driven by the large proportion of women in our study. Altogether, the increased T2D risks when substituting SFA by proteins, carbohydrates, and especially PUFA, needs to be interpreted with caution.

In conclusion, our findings emphasize the importance of distinguishing between the types and food sources of SFA in relation to T2D risk. Our study suggests that individual SFA that exist to a large extent in cheese, and SFA derived from cheese, relate to a lower T2D risk. Regarding substitution of SFA with other macronutrients, we cannot exclude the possibility that our results reflect inverse associations of dairy-derived fatty acids with T2D and/or residual confounding by *trans* fatty acid intake. Further investigation is still warranted, especially in populations with other dietary patterns contributing to SFA intake, and with more variation in intake of PUFA, to draw further conclusions of the relations of SFA with T2D risk.

## Electronic supplementary material

Below is the link to the electronic supplementary material.

Supplementary material 1 (DOCX 255 KB)
