# Supplementary material for: Intake of dietary saturated fatty acids and risk of type 2 diabetes in the European Prospective Investigation into Cancer and Nutrition-Netherlands cohort: associations by types, sources of fatty acids and substitution by macronutrients
Source: Eur J Nutr. 2018 Mar 9;58(3):1125–36. doi: 10.1007/s00394-018-1630-4 (PMC6499756; doi:10.1007/s00394-018-1630-4)
Supplement: Supplementary file 1 — Supplementary material 1 (DOCX 255 KB) [file 394_2018_1630_MOESM1_ESM.docx]

**Online Supplementary Material**


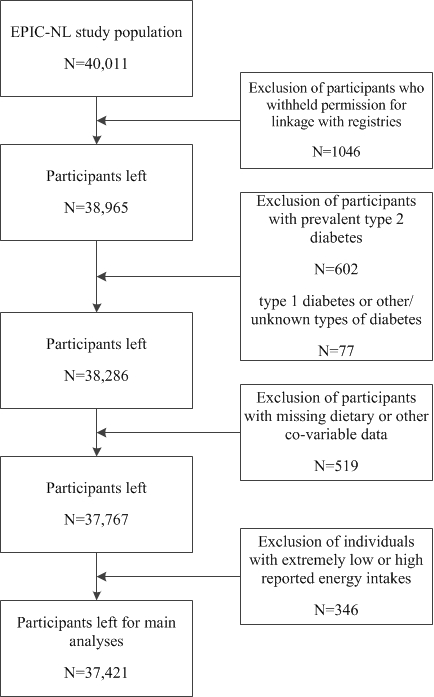


Online Supplemental Figure 1 Flow chart of the study population.


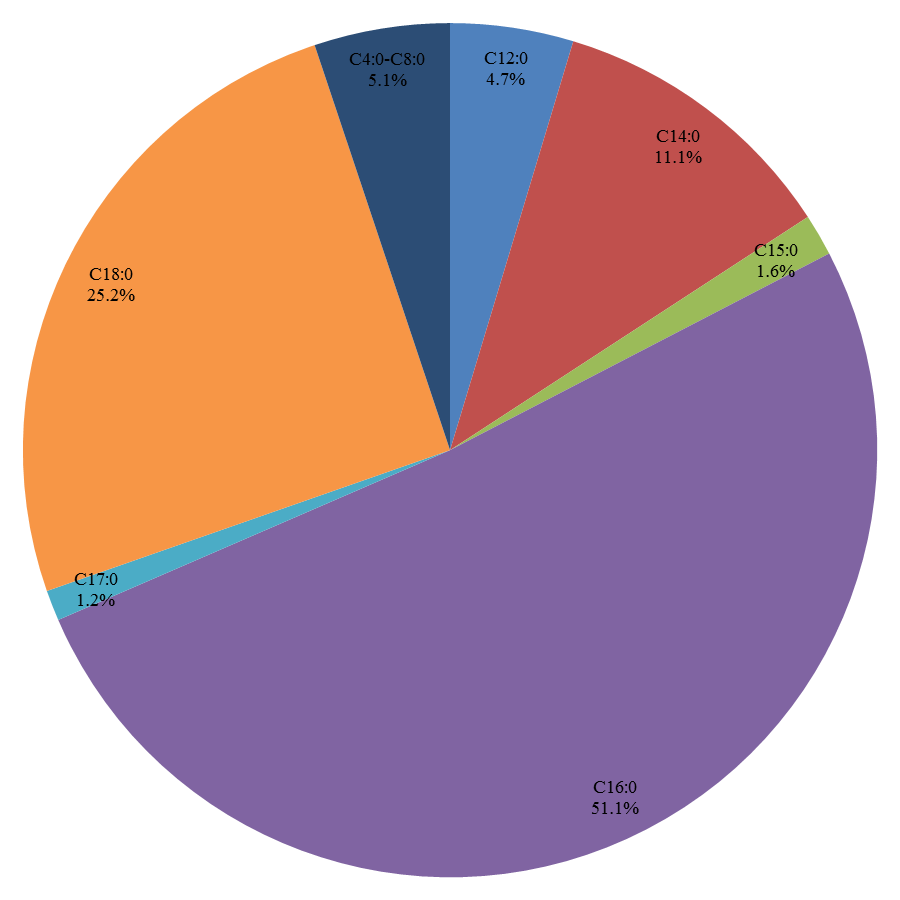


Online Supplemental Figure 2 Contributions (in percentages) of SFA types to the baseline total SFA intake in 37,421 participants of the European Prospective Investigation into Cancer and Nutrition–Netherlands cohort.


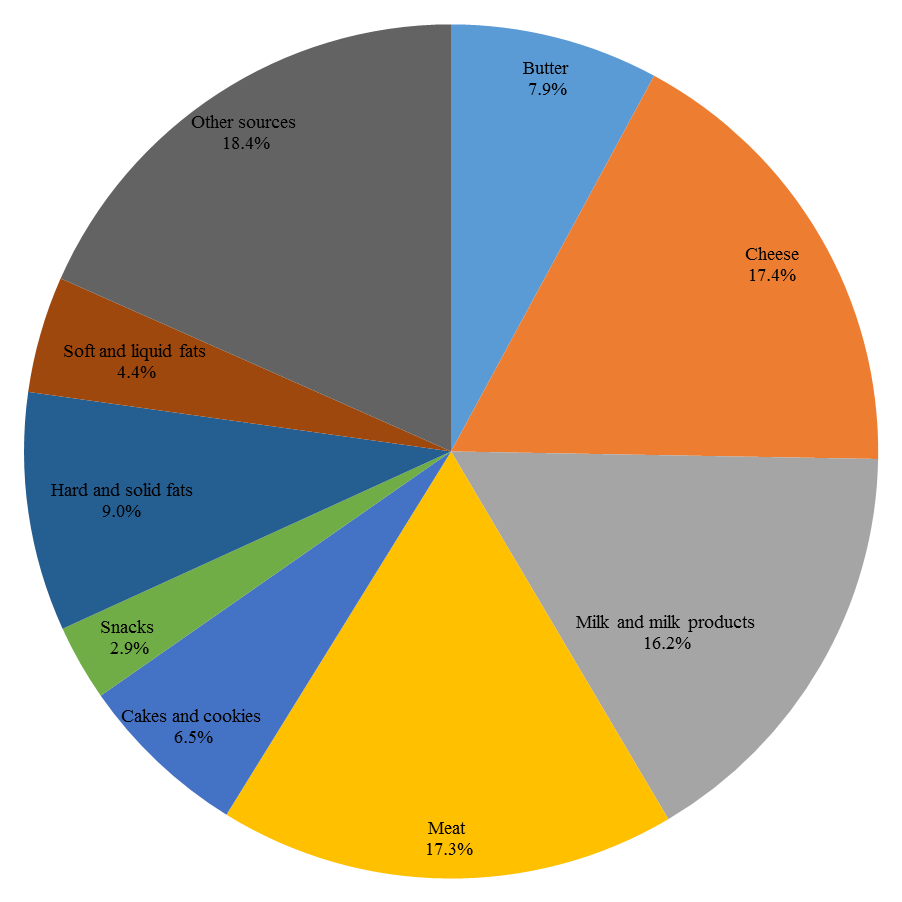


Online Supplemental Figure 3 Contributions (in percentages) of food groups to the baseline total SFA intake in 37,421 participants of the European Prospective Investigation into Cancer and Nutrition–Netherlands cohort.

**Online Supplemental Table 1** Pearson correlation coefficients between intake of SFA (en %) and specific SFAs in 37,421 participants from the EPIC-NL cohort ^a^

|  | (1) | (2) | (3) | (4) | (5) | (6) | (7) | (8) | (9) | (10) | (11) | (12) | (13) | (14) | (15) | (16) | (17) |
| --- | --- | --- | --- | --- | --- | --- | --- | --- | --- | --- | --- | --- | --- | --- | --- | --- | --- |
| (1) Total SFA | 1 |  |  |  |  |  |  |  |  |  |  |  |  |  |  |  |  |
| (2) Sum of butyric (4:0) to capric (10:0) acids | 0.57 | 1 |  |  |  |  |  |  |  |  |  |  |  |  |  |  |  |
| (3) Lauric acid (12:0) | 0.53 | 0.73 | 1 |  |  |  |  |  |  |  |  |  |  |  |  |  |  |
| (4) Myristic acid (14:0) | 0.80 | 0.84 | 0.71 | 1 |  |  |  |  |  |  |  |  |  |  |  |  |  |
| (5)Pentadecylic (15:0) | 0.67 | 0.94 | 0.59 | 0.92 | 1 |  |  |  |  |  |  |  |  |  |  |  |  |
| (6) Palmitic acid (16:0) | 0.87 | 0.39 | 0.31 | 0.67 | 0.54 | 1 |  |  |  |  |  |  |  |  |  |  |  |
| (7) Margaric acid (17:0) | 0.75 | 0.77 | 0.49 | 0.85 | 0.88 | 0.75 | 1 |  |  |  |  |  |  |  |  |  |  |
| (8)Sum of pentadecylic (15:0) and margaric(17:0) acids | 0.71 | 0.91 | 0.57 | 0.92 | 0.99 | 0.63 | 0.95 | 1 |  |  |  |  |  |  |  |  |  |
| (9)Stearic acid (18:0) | 0.82 | 0.29 | 0.31 | 0.54 | 0.41 | 0.92 | 0.65 | 0.50 | 1 |  |  |  |  |  |  |  |  |
| (10) SFA from Butter | 0.42 | 0.17 | 0.23 | 0.50 | 0.32 | 0.43 | 0.23 | 0.29 | 0.35 | 1 |  |  |  |  |  |  |  |
| (11) SFA from Cheese | 0.40 | 0.75 | 0.38 | 0.58 | 0.78 | 0.32 | 0.68 | 0.77 | 0.26 | 0.02 | 1 |  |  |  |  |  |  |
| (12) SFA from Milk and milk products | 0.23 | 0.47 | 0.46 | 0.45 | 0.39 | 0.07 | 0.28 | 0.36 | -0.02 | -0.04 | -0.11 | 1 |  |  |  |  |  |
| (13) SFA from Meat | 0.23 | -0.26 | -0.20 | -0.06 | -0.14 | 0.48 | 0.28 | 0.00 | 0.54 | -0.01 | -0.16 | -0.18 | 1 |  |  |  |  |
| (14) SFA from Cakes and cookies | 0.17 | 0.19 | 0.39 | 0.15 | 0.09 | 0.03 | 0.07 | 0.08 | 0.05 | -0.03 | 0.00 | 0.00 | -0.16 | 1 |  |  |  |
| (15) SFA from Snacks | -0.04 | -0.25 | -0.22 | -0.23 | -0.24 | 0.09 | -0.15 | -0.21 | 0.10 | -0.09 | -0.15 | -0.19 | 0.04 | -0.11 | 1 |  |  |
| (16) SFA from Hard, solid fats | 0.41 | -0.10 | 0.00 | 0.13 | -0.01 | 0.38 | 0.08 | 0.02 | 0.34 | 0.02 | -0.10 | -0.02 | 0.18 | -0.05 | -0.03 | 1 |  |
| (17) SFA from Soft, liquid fats | -0.12 | -0.11 | -0.09 | -0.18 | -0.14 | -0.14 | -0.15 | -0.14 | -0.13 | -0.08 | -0.03 | -0.11 | -0.03 | -0.08 | -0.09 | -0.20 | 1 |
| (18) SFA from Other sources | -0.03 | -0.32 | -0.23 | -0.31 | -0.32 | 0.02 | -0.30 | -0.32 | 0.16 | -0.06 | -0.19 | -0.27 | -0.16 | -0.05 | 0.32 | -0.11 | -0.07 |

^a^ All P values were <0.0001 unless stated otherwise. en%, percentage of energy. EPIC-NL, European Prospective Investigation into Cancer and Nutrition Netherlands.

**Online Supplemental Table 2** Pearson correlation coefficients between intake of SFA from different food source (g/d) and intake of total fatty acids from each food source (g/d) in 37,421 participants from the EPIC-NL cohort ^a^

|  | Fatty acids from Butter | Fatty acids from Cheese | Fatty acids from Milk and milk products | Fatty acids from Meat | Fatty acids from Cakes and cookies | Fatty acids from Snacks | Fatty acids from Hard, solid fats | Fatty acids from Soft, liquid fats | Fatty acids from Other sources |
| --- | --- | --- | --- | --- | --- | --- | --- | --- | --- |
| SFA from Butter | 1.00 |  |  |  |  |  |  |  |  |
| SFA from Cheese |  | 1.00 |  |  |  |  |  |  |  |
| SFA from Milk and milk products |  |  | 1.00 |  |  |  |  |  |  |
| SFA from Meat |  |  |  | 1.00 |  |  |  |  |  |
| SFA from Cakes and cookies |  |  |  |  | 0.99 |  |  |  |  |
| SFA from Snacks |  |  |  |  |  | 0.98 |  |  |  |
| SFA from Hard, solid fats |  |  |  |  |  |  | 0.97 |  |  |
| SFA from Soft, liquid fats |  |  |  |  |  |  |  | 0.94 |  |
| SFA from Other sources |  |  |  |  |  |  |  |  | 0.93 |

^a^ All P values were <0.0001 unless stated otherwise. EPIC-NL, European Prospective Investigation into Cancer and Nutrition Netherlands.

**Online Supplemental Table 3** Pearson correlation coefficients between intake of SFA from different food source (g/d) and intake of each food group (g/d) in 37,421 participants from the EPIC-NL cohort ^a^

|  | Butter | Cheese | Milk and milk products | Meat | Cakes and cookies | Snacks | Hard, solid fats | Soft, liquid fats | Other sources |
| --- | --- | --- | --- | --- | --- | --- | --- | --- | --- |
| SFA from Butter | 1.00 |  |  |  |  |  |  |  |  |
| SFA from Cheese |  | 0.98 |  |  |  |  |  |  |  |
| SFA from Milk and milk products |  |  | 0.79 |  |  |  |  |  |  |
| SFA from Meat |  |  |  | 0.92 |  |  |  |  |  |
| SFA from Cakes and cookies |  |  |  |  | 0.89 |  |  |  |  |
| SFA from Snacks |  |  |  |  |  | 0.92 |  |  |  |
| SFA from Hard, solid fats |  |  |  |  |  |  | 0.96 |  |  |
| SFA from Soft, liquid fats |  |  |  |  |  |  |  | 0.97 |  |
| SFA from Other sources |  |  |  |  |  |  |  |  | 0.22 |

^a^ All P values were <0.0001 unless stated otherwise. EPIC-NL, European Prospective Investigation into Cancer and Nutrition Netherlands.

**Online Supplemental Table 4** Multivariable HRs with 95% CIs for the association between the consumption of 1% of energy from different macronutrients at the expense of 1% of energy from total SFA while keeping total energy intake constant and incident type 2 diabetes risk in men and women of 37,421 participants from the EPIC-NL cohort ^a,b,c^

|  | Men | Women | P-value for interaction ^d^ |
| --- | --- | --- | --- |
| Vegetable protein | 1.22(0.97,1.53) | 1.12(0.99,1.26) | 0.195 |
| Animal protein | 1.00(0.91,1.09) | 1.13(1.08,1.18) | 0.059 |
| MUFA | 0.97(0.86,1.09) | 1.07(1.00,1.15) | 0.190 |
| PUFA | 0.91(0.73,1.13) | 1.18(1.05,1.32) | 0.066 |
| Carbohydrates | 0.97(0.92,1.02) | 1.06(1.03,1.10) | 0.013 |

^a^ Obtained from Cox proportional hazards regression models. EPIC-NL, European Prospective Investigation into Cancer and Nutrition–Netherlands.

^b^ Final adjusted model: adjustment for total energy intake; sex, age and sum of other SFA; education level, energy-adjusted alcohol consumption level, smoking status, physical activity level; energy-adjusted intake of vitamin E, dietary fibre and cholesterol; BMI and waist circumference.

^c^ HRs for each additional intake of 1% of energy from certain macronutrients at the expense of an equal amount energy from SFA.

^d^ P value was calculated from likelihood ratio test by including continuous interaction terms in the final adjusted model.

**Online Supplemental Table 5** Multivariable HRs with 95% CIs for the association between intake of non-SFA fatty acids from different food sources (en%) and incidence of type 2 diabetes in 37,421 participants from the EPIC-NL cohort ^a^

| Non-SFA fatty acids from: | Median intake en%/d | SD | HR (95% CI) |
| --- | --- | --- | --- |
| Butter | 0.35 | 0.76 | 0.93(0.86,1.01) |
| Cheese | 1.45 | 1.21 | 0.89(0.83,0.97) |
| Milk and milk products | 1.09 | 0.73 | 1.03(0.95,1.12) |
| Meat | 3.83 | 2.29 | 1.04(0.97,1.11) |
| Cakes and cookies | 0.80 | 0.81 | 0.97(0.90,1.05) |
| Snacks | 0.74 | 1.00 | 0.94(0.85,1.03) |
| Hard, solid fats | 1.42 | 1.74 | 0.94(0.87,1.02) |
| Soft, liquid fats | 2.49 | 2.20 | 1.07(0.97,1.17) |
| Others | 6.23 | 2.74 | 0.95(0.86,1.05) |

^a^ Obtained from Cox proportional hazards regression models. EPIC-NL, European Prospective Investigation into Cancer and Nutrition–Netherlands. HRs are expressed per SD en%. Non-SFA fatty acids from different food source entered the models as a continuous variable per SD of intake (en %), and were adjustedt for total energy intake; sex, age and sum of other non-SFA fatty acids; education level, energy-adjusted alcohol consumption level, smoking status, physical activity level; energy-adjusted intake of animal protein, vegetable protein, *trans* fatty acids, vitamin E, dietary fibre and cholesterol; BMI and waist circumference.
